# Comparative proton and photon treatment plans in children treated for neuroblastoma

**DOI:** 10.2340/1651-226X.2025.43865

**Published:** 2025-07-23

**Authors:** Anna Embring, Ingrid Kristensen, Martin P. Nilsson, Jacob Engellau, Malin Blomstrand, Charlotta Fröjd, Måns Agrup, Anna Flejmer, Anna-Maja Svärd, Anna Asklid

**Affiliations:** a Department of Oncology, Karolinska University Hospital, Stockholm, Sweden; b Department of Oncology-Pathology, Karolinska Institute, Stockholm, Sweden; c Department of Hematology, Oncology and Radiation Physics, Skane University Hospital, Lund, Sweden; d Department of Oncology, Clinical Sciences, Lund University, Lund, Sweden; e Department of Oncology, Sahlgrenska University Hospital, Gothenburg, Sweden; f Department of Oncology, Institute of Clinical Sciences, University of Gothenburg, Sweden; g Department of Oncology, and Department of Biomedical and Clinical Sciences, Linköping University, Linköing, Sweden; h Department of Immunology, Genetics and Pathology, Uppsala University, Uppsala, Sweden; i Department of Radiation Sciences, Oncology, Umeå University, Sweden

**Keywords:** Neuroblastoma, paediatrics, radiotherapy, protons, photons, adverse effects

## Abstract

**Background and purpose:**

Neuroblastoma is the most common extracranial solid tumour in children. Radiotherapy is commonly part of the multimodal treatment for high-risk patients. The aim of this study is to analyse doses to organs at risk (OAR) in comparative proton and photon treatment plans for children treated for neuroblastoma and report side effects.

**Patient/material and methods:**

All children in Sweden treated with curative intent radiotherapy for abdominal neuroblastoma in 2017–2024 with comparative proton and photon treatment plans were retrospectively identified through a national registry (RADTOX), where data on side effects were collected. Doses to OAR were compared in each patient’s proton and photon treatment plans.

**Results:**

A total of 30 children with a median age of 45 months (range 11–150) were included. The low-dose spread was significantly lower in the proton compared to the photon treatment plans measured as Body V5Gy and V10Gy (*p* < 0.001). Furthermore, the mean doses to the bowel bag, kidneys, liver, pancreas, and spleen were significantly lower in the proton plans. The median follow-up was 14 months (1–61), and the 2-year overall survival was 75.3%. While acute radiotherapy related grade ≥ 2 side effects were experienced by 12 patients (40%), late side effects were experienced by 7 patients (13%). The most common side effects were haematological and from the upper gastrointestinal tract.

**Interpretation:**

In selected cases, proton treatment can offer lower doses to OAR and less low-dose exposure compared to photon treatment in children treated for abdominal neuroblastoma. Whether this translates into a clinical benefit is currently unclear and should be evaluated in future studies.

## Introduction

Neuroblastoma is the most common type of extracranial solid tumour in children, with about 20 new cases diagnosed annually in Sweden [1]. The tumour arises from the sympathetic nervous system, and more than half of neuroblastomas originate from the adrenal glands [2]. Neuroblastoma predominantly affects children under the age of 4 [3], and the prognosis varies significantly based on tumour biology, ranging from a 5-year survival rate exceeding 95% in low-risk neuroblastoma, to approximately 50% in children with high-risk disease [2]. The prognosis for children with high-risk neuroblastoma has markedly improved over recent decades due to the implementation of multimodal treatment strategies, including chemotherapy, surgical resection, high-dose chemotherapy with autologous stem cell rescue, radiotherapy, and immunotherapy [4]. However, the intensification of treatment regimens has led to an increase in long-term health complications. According to the Childhood Cancer Survivor Study (CCSS), neuroblastoma survivors have a 20-year cumulative incidence of chronic health conditions of 41.1%, with those receiving multimodal treatment being more than twice as likely to develop chronic health issues compared to those treated with surgery alone [5]. The same study reports that secondary malignancy is a common cause of late mortality, second only to recurrent disease. The Pediatric Normal Tissue Effects in the Clinic (PENTEC) report on subsequent neoplasms in childhood cancer survivors after radiation therapy shows a significant dose-response relationship between radiation exposure and the development of secondary malignancies, advocating for the use of conformal radiotherapy techniques [6].

The risk of severe late side effects of radiotherapy is correlated to younger age, higher radiation doses, and larger treatment volumes [7]. Given that neuroblastoma often affects very young children and requires relatively large treatment volumes, the risk of radiation-induced late effects is substantial, necessitating measures to minimise these risks. Proton radiotherapy has been shown to enable lower doses to organs at risk (OAR) compared to photon radiotherapy [8]. Despite this, proton radiotherapy is not yet the standard of care for treating paediatric neuroblastoma.

In Sweden, all paediatric radiotherapy centres have access to proton radiotherapy through the national proton centre, the Skandion Clinic, which opened in 2015 and began treating children in 2016. Proton radiotherapy is fully subsidised by the government, covering treatment, travel, and accommodation costs for families. The decision to use proton radiotherapy is made by the treating radiation oncologist based on the potential benefits for the patient. All patients referred to the Skandion Clinic undergo comparative proton and photon treatment planning, reviewed at a national video conference where the treatment modality is decided by consensus, considering dosimetric, clinical, social, and other factors. This routine provides a unique opportunity to analyse dosimetric differences between proton and photon treatment plans in clinical practice.

The aim of this retrospective study is to analyse doses to OAR, comparing proton and photon treatment plans, to investigate potential dosimetric advantages of each modality, and to report associated side effects in children treated for neuroblastoma with radiotherapy.

## Patients/material and methods

**Figure 1:**
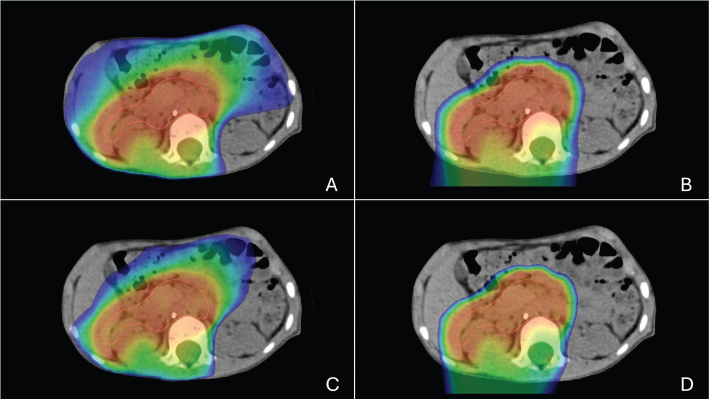
Dose distributions in colour wash of the volume that received ≥ 5 Gy (V5Gy) and ≥ 10 Gy (V10Gy). (A) photon treatment and V5Gy; (B) proton treatment plan and V5Gy; (C) photon treatment and V10Gy; (D) proton treatment plan and V10Gy.

All paediatric patients in Sweden treated with curative intent radiotherapy for abdominal neuroblastoma between 2017 and 2024, with comparative proton and photon treatment plans, were included in this study. The clinical decision to evaluate proton radiotherapy for the individual patient was made by the treating radiation oncologist. Patients were identified through RADTOX, a prospectively maintained national registry for paediatric radiotherapy, where data on side effects are recorded. Side effects were classified as acute if they occurred during radiotherapy or within 3 months post-treatment, and as late if presenting thereafter. Side effects were graded according to the Radiation Therapy Oncology Group (RTOG) and the European Organization for Research and Treatment of Cancer (EORTC) score [9], and were reported in the study if assessed to have definitive/most probable relation to radiotherapy or assessed being caused by a combination of reasons. Side effects assessed to have unlikely or no relation to radiotherapy were not included. Dosimetric data were extracted from clinical treatment plans, with low-dose spread defined as the percentage of the scanned body receiving ≥ 5 Gy (Body V5Gy) and ≥10 Gy (Body V10Gy) (Figure 1). A conformity index was calculated as the volume receiving 95% of the prescribed dose divided by the clinical target volume (CTV), facilitating evaluation of conformity between radiotherapy modalities.

Target delineation was performed according to local clinical practice at the time, mainly in accordance with the SIOPEN HR-NBL-1 protocol [10]. Variations in target delineation, however, did not impact the study results, as each patient served as their own control, with identical CTV and OAR used in both proton and photon treatment plans. Patients lacking delineation of specific OAR were excluded from the comparative dose analysis for those particular OAR.

The study was approved by the National Ethical Review Authority (Dnr 2024-03803-01), and results are reported following the STROBE cohort reporting guidelines [11].

### Proton radiotherapy at the Skandion Clinic

The Skandion Clinic is a pencil beam scanning facility equipped with two gantries, both featuring orthogonal X-ray imaging, with one gantry in addition equipped with cone-beam computed tomography (CBCT). Daily imaging, either orthogonal X-ray or CBCT, is performed to ensure precise patient positioning. In addition, weekly computed tomography (CT) scans are conducted to assess anatomical changes and verify target coverage. Beam delivery is performed with energies ranging from 60 to 226 MeV (Ion Beam Applications, Belgium). For treatment planning, robust optimisation is performed with a range uncertainty of 3.5% and a set-up uncertainty of 5–7 mm. Robustness evaluation mandates that 98% of the CTV must be covered by at least 95% of the prescribed dose for at least 12 of the 14 uncertainty curves. Proton treatment planning is conducted using Varian Eclipse software (Varian Medical Systems, Palo Alto, California). All clinical proton plans undergo measurement and validation at the Skandion Clinic prior to the start of treatment. In addition, a CT scan is performed at the clinic before treatment initiation to evaluate dose distribution and validate the treatment plan.

### Statistical analysis

Overall survival (OS) was estimated using the Kaplan-Meier method, with OS defined as the interval from the last day of radiotherapy to either death or the last follow-up date, whichever occurred first. Doses to OAR were compared in each individual patient’s clinical proton and photon treatment plans, and differences were tested through Wilcoxon signed-rank test. The association between side effects and treatment modality was evaluated using the chi-square test. IBM SPSS® Statistics v29.0.1.0 was used for statistical analysis, with a significance threshold set at *p* < 0.05. Box plot calculations were executed using Microsoft Excel.

## Results

**Figure 2:**
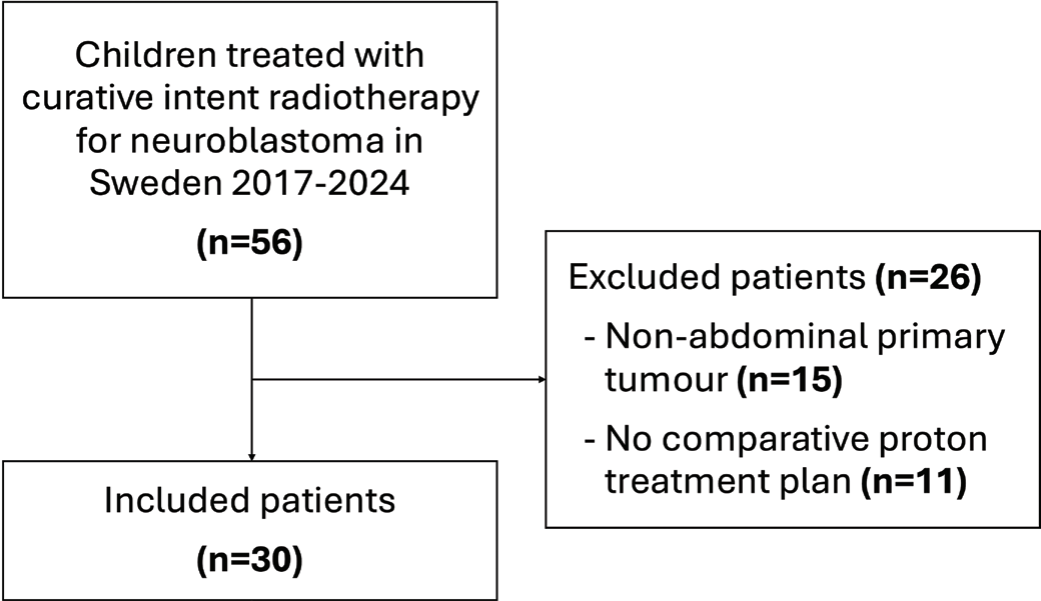
Flow chart of included patients. n – number of patients.

A total of 56 paediatric patients with neuroblastoma were treated with curative intent radiotherapy between 2017 and 2024 in Sweden. Among these, 41 had tumours located in the abdomen, and 30 had comparative proton and photon treatment plans created and were included in the study (Figure 2). The median age of the included patients at the start of radiotherapy was 45 months (range 11–150), with an equal distribution of females (50%) and males (50%). A majority (90%) received 21 Gy in 14 fractions, while the remaining 10% received 36 Gy in 18–22 fractions (21 Gy + sub-volume boost to 36 Gy). The median follow-up was 14 months (range 1–61), and the 2-year OS was 75.3% (Figure S1). Seven patients died of neuroblastoma and one patient died of unknown causes.

In the studied cohort, comparative proton and photon treatment plans were created for all patients. Proton therapy was selected for 21 patients and photon therapy for 9 patients. The most common reasons for choosing treatment with photons over protons were equal treatment plans (*n* = 4), and uncertainties in the proton plans due to air/movement (*n* = 3). Furthermore, one patient was treated with photons because of uncertainties due to metal close to the target and another patient because of logistical reasons (in poor general condition and was treated at the intensive care unit). Among the nine patients treated with photons, eight received volumetric-modulated arc therapy (VMAT), and one received 3D-conformal technique.

**Figure 3:**
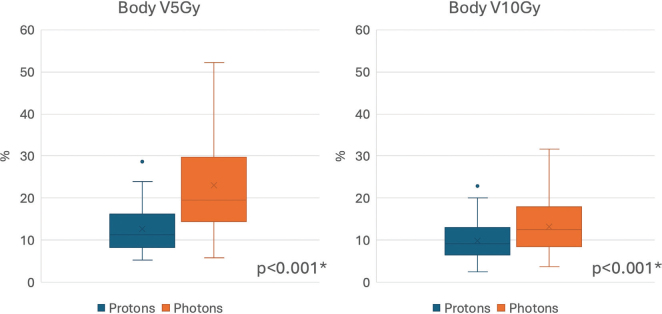
Low-dose spread shown as the percentage of the scanned body that received ≥ 5 Gy (Body V5Gy) and ≥ 10 Gy (Body V10Gy) in comparative proton and photon treatment plans. *indicating statistically significant difference.

**Figure 4:**
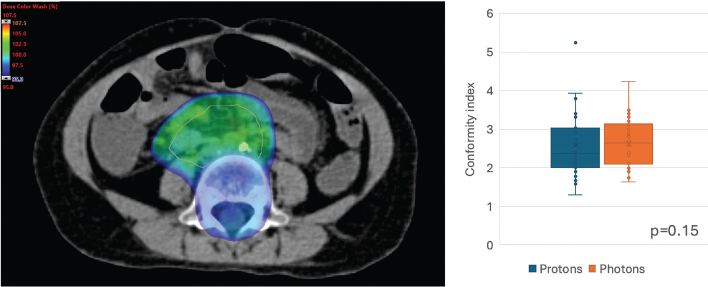
Conformity index defined as the volume that have received 95% of prescribed dose divided by the clinical target volume (CTV). Image showing clinical example of a neuroblastoma treatment plan with a CTV (orange line) and volume receiving ≥ 95% of prescribed dose in colour wash. Box plots showing conformity index compared in proton and photon treatment plans.

Comparative analysis of proton and photon treatment plans for each patient showed that the low-dose spread, measured by Body V5Gy and V10Gy, was significantly lower with protons (*p* < 0.001) (Figure 3). There was no significant difference in conformity index (volume receiving 95% of the prescribed dose divided by the CTV) between proton (Md = 2.37, IQR 2.01–3.04) and photon (Md = 2.63, IQR 2.09–3.13) treatment plans, *p* = 0.15 (Figure 4).

**Figure 5:**
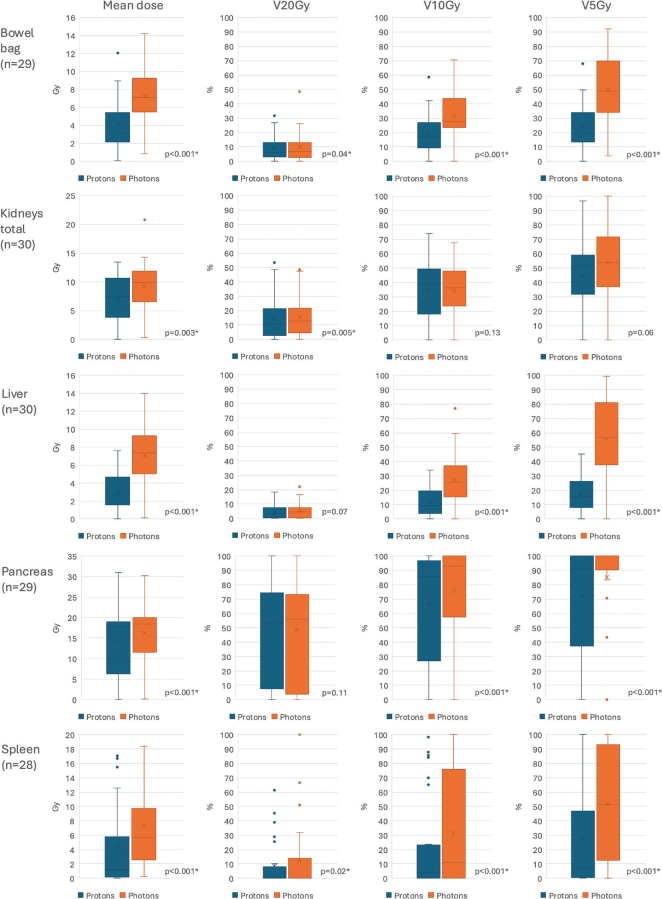
Boxplots showing doses to organs at risk in comparative proton and photon treatment plans. *indicating statistically significant difference. n – number of patients.

The mean doses to all investigated OAR (bowel bag, kidneys, liver, pancreas, and spleen) were significantly lower in the proton treatment plans compared to the photon treatment plans (Figure 5 and supplementary material Table S1). The volume in each OAR that received ≥ 5 Gy, ≥ 10 Gy and ≥ 20 Gy are reported in Figure 5.

While 12 patients (40%) experienced any radiotherapy related grade ≥ 2 acute side effect, 7 patients (13%) experienced any radiotherapy related grade ≥ 2 late side effect. The most common types of both acute and late side effects were upper gastrointestinal tract side effects and haematological toxicity (Table 1). For acute side effects 75% were assessed to be caused by a combination of reasons (including radiotherapy) and 25% were assessed to have a definitive relation to given radiotherapy. For late side effects all were assessed to be caused by a combination of reasons. There was no significant correlation between side effects and treatment modality.

**Table 1:** Side effects with possible relation to radiotherapy.

Type of side effect	Acute		Late	
	Number (%)		Number (%)	
	Grade ≥ 2	Grade ≥ 3	Grade ≥ 2	Grade ≥ 3
Blood tests	5 (17)	4 (13)	6 (20)	1 (3)
Upper gastrointestinal tract	7 (23)	6 (20)	2 (7)	2 (7)
Liver function tests	1 (3)	0 (0)	0 (0)	0 (0)
General condition	1 (3)	0 (0)	0 (0)	0 (0)
Peripheral nerves	0 (0)	0 (0)	1 (3)	1 (3)
Function of irradiated extremity	0 (0)	0 (0)	1 (3)	0 (0)

## Discussion and conclusion

To our knowledge, this is the largest study comparing treatment plans for pencil beam scanning proton therapy to modern photon radiotherapy in children with neuroblastoma. Our findings indicate that proton radiotherapy has the potential to reduce radiation dose across a wide range of OAR when treating abdominal neuroblastoma, which aligns with previously published results. For instance, a dosimetric study involving 14 patients with abdominal neuroblastoma reached similar conclusions regarding the ability of proton radiotherapy to spare OAR [12]. Several studies have demonstrated that even low or moderate radiation doses can cause side effects in the paediatric population. In the PENTEC report on kidney disease in childhood cancer survivors, whole kidney exposure of 10.2 Gy and 14.5 Gy was associated with a 5% risk of grade ≥ 2 and grade ≥ 3 renal toxicity, respectively [13]. Data on partial liver irradiation are limited, but the PENTEC report on liver suggests that whole liver radiotherapy < 10 Gy is associated with a ≤6% risk of developing sinusoidal obstructive syndrome [14]. The CCSS study on diabetes mellitus in childhood cancer survivors revealed a 6.9-fold increased risk of developing diabetes mellitus after abdominal radiotherapy for neuroblastoma, an increased risk not observed in neuroblastoma patients treated with non-radiotherapy regimens [15]. A review article by Hessels et al. recommends aiming for ≤10 Gy to the pancreas to minimise the risk of developing diabetes mellitus [16]. The European Society for Paediatric Oncology (SIOPE) Radiation Oncology Working Group advises a mean dose to the spleen < 10 Gy, if possible, without compromising target coverage, to reduce the risk of overwhelming post-splenectomy infection [17]. Altogether, these findings support the goal of reducing low-dose exposure to surrounding healthy tissue when treating children with radiotherapy.

The most commonly reported late side effects following neuroblastoma treatment are endocrine, sensory (including hearing loss), dental, musculoskeletal, neurological, and neurocognitive effects [5, 18–20]. Depending on the location of the tumour, radiotherapy could impact all these systems. However, since the most common location of neuroblastoma is the abdomen, radiotherapy predominantly affects musculoskeletal, endocrine (due to irradiation of the pancreas), and secondary malignancies. The PENTEC-report on subsequent neoplasms shows a significant radiation dose-response relationship for secondary neoplasms and hypothesise that radiation techniques with improved conformality could reduce the risk of secondary neoplasms after radiotherapy [6]. Similarly, Taylor et al. demonstrated that pencil beam scanning proton therapy was associated with lower risk of radiation-induced secondary malignancies compared to photon treatment in the treatment of neuroblastoma in the abdomen, as estimated using a risk model [21].

In the current study, the most common side effects were upper gastrointestinal tract side effects and haematological toxicity. The incidence of radiotherapy-related side effects was higher compared to previously published results. In the report from the German KiProReg Registry [22] a total of 44 patients treated with proton radiotherapy for neuroblastoma were included and only 16% experienced grade ≥ 3 acute side effects, compared to 30% in the current study. In the retrospective study by Hill-Kayser et al. including 45 high-risk neuroblastoma patients treated with proton radiotherapy, acute toxicities were mainly grade 1 and no patients experienced significant radiotherapy-related late toxicity [23]. One reason for this difference could be the relatively small sample size of our cohort to evaluate side effects. Another could be that we reported all side effects assessed as due to multiple reasons as radiotherapy-related, whereas other studies may have more strict definitions on which side effects should be considered to be caused by radiotherapy.

Intensified treatment of high-risk neuroblastoma has resulted in improved survival but also in more late side effects. This is believed to be the reason why neuroblastoma survivors are among the few groups that have experienced an increase in late mortality after treatment in recent decades compared to earlier reports [5]. In accordance with this, the newly published study on long-term outcomes and quality of life in patients treated for high-risk neuroblastoma 1989–2002 in Germany confirms that late side effects are common in this population, with all (*n* = 25, 100%) experiencing late side effects [24]. However, reported quality of life was better across multiple scales compared to the general public.

There was no significant difference in the conformity index between proton and photon treatment plans. This is not unexpected, as both treatment modalities can be considered conformal techniques regarding the high-dose volume. However, in both proton and photon treatment plans, the conformity index could be considered somewhat high, with the volume receiving 95% or more of the prescribed dose being twice or three times the size of the CTV in most treatment plans. This is likely due to the localisation of abdominal neuroblastomas and the proximity to the vertebrae, necessitating the inclusion of adjacent vertebrae to mitigate the development of scoliosis [25].

The proton treatment plan of one of the included patients is an outlier with considerably higher doses to OAR compared to the other treatment plans. Upon closer investigation, this treatment plan was not fully optimised due to an early decision to treat with photons due to metal near the target and the proton plan was not fully finalised. A more common reason for choosing to treat with photon radiotherapy in the current study was uncertainties in the proton treatment plans due to air/movement. The uncertainties due to the interplay effect using pencil beam scanning in paediatric neuroblastoma was investigated by Lim et al. in a study including 19 patients with a median age of 3.5 years [26]. They found that the organ movements were generally small, with a mean maximum cranio-caudal movement of 2.7 mm, and even smaller movements in other directions. They concluded that even though volumetric rescanning is feasible as motion mitigation, the clinical relevance is debatable with < 5 mm organ motion.

In the current study, the majority of all patients were treated with 21 Gy; but 10% were treated with a boost to residual tumour to a total dose of 36 Gy. The recently published retrospective study by Samim et al. shows promising results of locoregional control after treating residual tumour ≥ 1 cm^3^ with a boost to a total dose of 36 Gy [27]. The value of adding a radiotherapy boost to residual tumour in neuroblastoma is also investigated in the ongoing randomised, international, and multicentric phase 3 study — SIOPEN HR-NBL-2 (EudraCT 2019-001068-31). There is a possibility that we will see a higher standard radiation dose in the future to neuroblastoma patients with residual tumour after surgery or when surgery is considered unsafe. An escalation in radiation dose in this often very young population calls for careful risk/benefit assessment, where choice of treatment modality may play an important role.

Although patients were treated at different centres and sometimes with different radiation doses, a notable strength of this study is that each patient served as their own control. In addition, the collection of side effects from the national registry (RADTOX) with prospectively gathered data enhance the robustness of the findings. However, there are some limitations to consider. Despite demonstrating dosimetric advantages for proton treatment compared to photon treatment, it remains unclear whether these advantages translate into improved patient outcomes, and this should be evaluated in future studies. The limited follow-up period is insufficient to investigate secondary malignancies or late bowel toxicity. Furthermore, only patients with comparative proton and photon treatment plans were included, which could be a source of selection bias, as no patients treated with photon treatment without a comparative proton treatment plan were included. There is a possibility that the treating radiation oncologist could foresee which patients were more likely to benefit from treatment with protons and hence, had comparative treatment plans calculated for these patients only. Nevertheless, given that this study includes neuroblastoma patients of various ages from all paediatric radiotherapy centres in Sweden and demonstrates dosimetric advantages for proton treatment, it is reasonable to recommend the use of comparative proton and photon treatment plans. This approach would enable the optimal selection of treatment modality and individualised radiotherapy for neuroblastoma patients.

In conclusion, for selected paediatric patients undergoing radiotherapy for abdominal neuroblastoma, proton therapy offers significant advantages over photon therapy by delivering lower doses to OAR and reducing low-dose exposure. This study underscores the importance of generating comparative proton and photon treatment plans for children with neuroblastoma to enable optimal treatment modality selection. The most frequently reported side effects were haematological and upper gastrointestinal tract toxicities.

## Supplementary Material



## Data Availability

Research data are stored in an institutional repository and will be shared upon reasonable request to the corresponding author.
