# Supplementary material for: Comparative proton and photon treatment plans in children treated for neuroblastoma
Source: Acta Oncol. 2025 Jul 23;64:43865. doi: 10.2340/1651-226X.2025.43865 (PMC12305685; doi:10.2340/1651-226X.2025.43865)
Supplement: Supplementary file 1 [file AO-64-43865-s1.pdf]

Supplementary material has been published as submitted. It has not been copyedited, or typeset by Acta Oncologica

Supplementary material

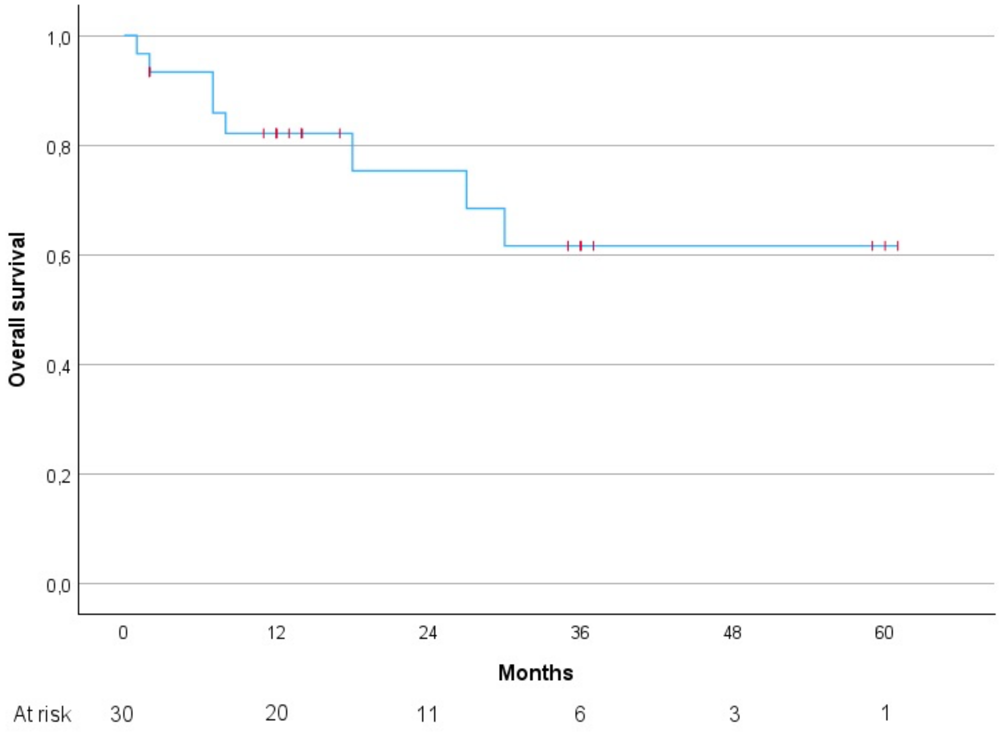

Figure S1: Kaplan-Meier estimate of overall survival in the total cohort. Solid blue curve is the Kaplan-Meier estimator, red vertical markers show censored patients.

Table S1: Doses to organs at risk

Organ at risk	Median mean dose in Gy (IQR)		*p*-value
	Proton treatment plan	Photon treatment plan	
Bowel bag	3.74 (2.16-5.45)	7.15 (5.49-9.27)	$p < 0.001$
Kidneys (total)	7.39 (3.83-10.65)	9.90 (6.54-11.92)	$p = 0.003$
Liver	2.73 (1.58-4.67)	7.38 (5.03-9.29)	$p < 0.001$
Pancreas	17.89 (6.36-19.01)	18.45 (11.53-20.09)	$p < 0.001$
Spleen	1.19 (0.14-5.80)	5.67 (2.56-9.74)	$p < 0.001$

IQR – interquartile range
